# Serum multi-cytokines screening identifies TRAIL and IL-10 as probable new biomarkers for prostate health index diagnostic utility adjustment in grey zone aggressive prostate cancer detection: A single-center data in China

**DOI:** 10.3389/fimmu.2022.901176

**Published:** 2022-08-18

**Authors:** Haojie Chen, Jiatong Zhou, Jia Luo, Yanyuan Wu, Yuhang Qian, Yuntian Shi, Fajun Qu, Bowen Shi, Jie Ding, Xingang Cui, Yongjiang Yu

**Affiliations:** ^1^ Department of Urology, School of Medicine, Xinhua Hospital Affiliated to Shanghai Jiao Tong University, Shanghai, China; ^2^ Department of Urology, Shanghai Children’s Hospital, Shanghai Jiao Tong University, Shanghai, China; ^3^ Department of Ophthalmology, School of Medicine, Xinhua Hospital Affiliated to Shanghai Jiao Tong University, Shanghai, China; ^4^ Department of Urology, Huadong Hospital Affiliated to Fudan University, Shanghai, China

**Keywords:** prostate cancer, PSA grey zone, prostate health index, TRAIL, IL-10

## Abstract

**Objective:**

To identify less invasive and easily applicable serum cytokine-derived biomarkers which contribute to the diagnostic utility and risk assessment ability of the prostate health index (PHI) based multivariable model in grey zone aggressive prostate cancer (AG PCa) early detection.

**Methods:**

Serum 45 cytokines screening was performed in a small training cohort consisting of 10 sera by Luminex liquid array-based multiplexed immunoassays and identified TRAIL and IL-10 as new biomarkers for PHI diagnostic utility adjustment for further validation with a multivariable predictive model in a cohort including 79 aggressive prostate cancer patients and 209 benign prostatic hyperplasia or indolent PCa patients within the PSA grey zone.

**Results:**

TRAIL and IL-10 were identified as potential serum biomarkers for AG PCa detection by the result of multi-cytokines screening in the univariate analysis, while multivariable logistic regression confirmed the AUC of the full risk predictive model (0.915) including tPSA, fPSA, PHI, TRAIL, and IL-10 was higher than various diagnostic strategies. DCA suggested a superior net benefit and indicated a good discriminative ability of the full risk model consistently with the result of the nomogram.

**Conclusion:**

We suggest a significant advantage for the PHI-based multivariate combinations of serum TRAIL and IL-10 comparing to PHI or other serum-derived biomarkers alone in the detection and risk stratification of grey zone AG PCa.

## Introduction

Prostate cancer (PCa), as one of the most malignant causes of cancer-related death in America, has an increasing incidence in Asia, especially in China with a rapidly aging society (1, 2). Early detection of aggressive PCa (AG PCa) may offer opportunities to revolutionize the survival benefits of this occult onset lethal disease and be regarded as a promising strategy aiming at cancer control (3). Currently, the only widely-used routine clinical screening biomarker for PCa is serum prostate-specific antigen (PSA), which has been garnering the criticism of low diagnostic specificity (25–40%) within the so-called “PSA grey zone” and resulting in a substantial increase in benign prostatic hyperplasia (BPH) or clinically indolent disease unnecessary biopsies (4, 5). The majority of biopsies cases trigged by moderately elevated serum total PSA levels are eventually proven to be indolent diseases that may not require active clinical intervention (6). It is thus urgent to precisely recognize those AG PCa at greater risk by detection methods with higher specificity and mitigate overdiagnosis of BPH or indolent disease, so that access to public medical resources may be allocated more appropriately.

Methods to improve risk stratification include the comprehensive utilization of the clinical information available and the improvement of novel biomarkers that are more specific for AG PCa detection (7, 8). To achieve this purpose, novel serum-based predictors have been emerging including [−2] pro-prostate specific antigen (p2PSA), %p2PSA, and prostate health index (PHI) in the past decade (9–11). Among them, PHI score, being a continuous variable, has been considered to possess superior predictive accuracy comparing with other PSA-derives. PHI can be used to direct the decision for prostate biopsy by choosing the best threshold with an associated sensitivity and specificity (12). Despite this, limitations of the crude risk stratification by PHI are disclosed when being validated in external cohorts consisting of different races/ethnicity of participants, populations, and genetic properties (3). Moreover, recent studies have reported the utility of the imaging assessment from TRUS and mpMRI (13, 14). PHI density (PHID) was developed combining with PHI and total prostate volume (15). However, the additional predictive value from PHID remained unclear, for extra clinical exams might reduce participation in early screening and increase the risk of bias of volume measurement from different ultrasonologists (16). Thus, appropriate non-invasion modification aiming to the better diagnostic utility of PHI for grey zone aggressive prostate cancer detection is warranted due to the highly heterogeneous and large individual differences among patients.

Recent studies have reported that tumor heterogeneity plays an essential role in tumor immune responses, contributing to tumorigenesis (17). Our previous work has described the correlation between elevated levels of serum cytokines IL-6 and TNF-α in the cancer progression and grading changes in localized PCa (18), showing the great potentiality of cytokines to impact cancer diagnostics. Yet data on grey zone AG PCa identification remain limited. There are several biases for single cytokine studies, which lead to the non-comprehensive understanding of cytokines’ real role in tumor diagnosis and limit their generalizability (19–22). Furthermore, the progression of PCa is a multistep process involving several growth factors, hormones, and cytokines. Thus, a multi-cytokine screening focusing on grey zone AG PCa discrimination is urgently needed to identify malignant-related cytokines which contribute to the diagnostic ability of PHI-based risk stratification.

This study aimed to perform a serum multi-cytokine screening to evaluate the plasma concentrations of 45 cytokines in grey zone AG PCa patients comparing with indolent/benign controls and identify novel candidates which contribute to PHI-based multivariate models’ diagnostic utility adjustment.

## Methods

### Study population

This was a cross-sectional study conducted on 320 senile Chinese patients with suspicious PCa who underwent initial biopsies due to elevated total PSA levels (4.0–10.0 ± 5% ng/mL) without DRE abnormal findings from August 2020 to March 2022. Men with a previous history of PCa, 5-α reductase inhibitors treatment, inability to sign informed consent, or the records of any serum antigen level were missing were excluded. The final cohort consisted of 288 patients (90%) including 79 aggressive PCa (AG PCa) and 209 indolent PCa or BPH after getting written informed consent. All patients underwent a biopsy guide by TRUS with at least 12 cores with or without radical prostatectomy after collecting blood samples for the PHI test and serum cytokine detection. Pathology outcomes from systematic prostate biopsies were considered the gold standard. Prostate biopsy specimens were analyzed and graded by experienced and skilled pathologists according to the 2014 International Society of Urological Pathology Consensus Conference (23). Aggressive prostate cancer (AG PCa) was defined as ≥Gleason 7 while indolent PCa as Gleason 6.

### Laboratory methods

After obtaining written informed consent, 8ml of blood was collected and kept in a vacutainer tube from each study participant and transported within 30min to the laboratory for processing. The serum was isolated by centrifuging blood at 1500g for 10 min at room temperature and stored at -80°C after centrifugation. The serum samples were anonymized and aliquoted before storage.

### Luminex cytokine immunoassays

**Figure 1:**
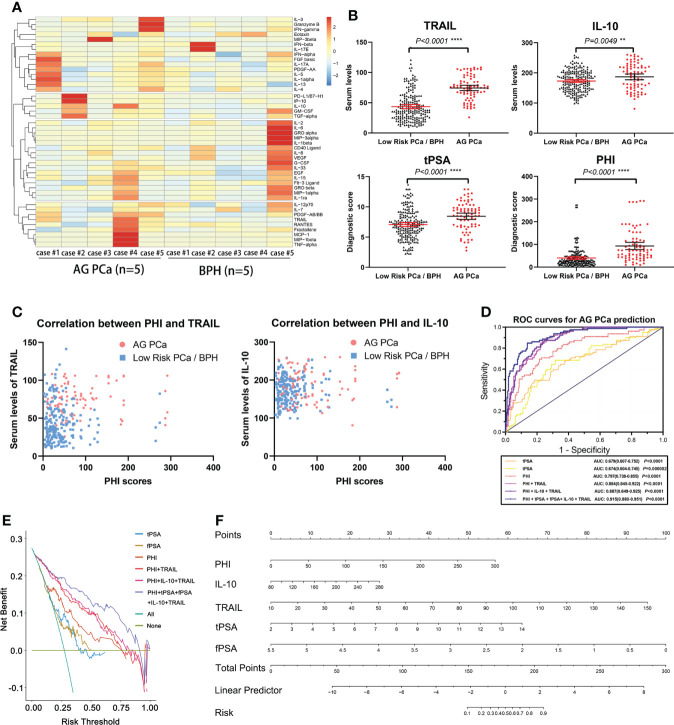
**(A)** Heatmap of differentially expressed 45 serum multi-cytokines between individuals with BPH and AG PCa using Luminex immunoassays testing. **(B)** Analysis of serum levels and diagnostic scores from indolent PCa/BPH and AG PCa patients. TRAIL, IL-10, tPSA, and PHI are demonstrated in scatterplots. *, *P*<0.05; **, *P <*0.01; ***, *P <*0.001; ****, *P <*0.0001 **(C)** Correlation scatter plots graph of PHI with TRAIL (left) and IL-10 (right). Outliers were found not to affect the significance of the results. **(D)** Receiver-operating analysis (ROC) curve of multivariate evaluation models for AG PCa prediction. **(E)** Decision-curve analysis for the performance of prediction models for AG PCa. **(F)** Nomogram predicting aggressive prostate cancer (AG PCa). Instructions: Each variable is located on its axis. Draw each vertical line upward to the “points” axis to determine the number of points attributed to each variable. Sum the points for each variable and locate them on the “total points” axis. Draw a vertical line down to find the probability of AG PCa. tPSA, total PSA; fPSA, free PSA; p2PSA, [-2] pro-PSA; PHI, prostate health index; IL-10, interleukin 10; TRAIL, TNF-related apoptosis induced ligand.

Stored serum samples were retrieved and thawed at 4°C. For the discovery stage, human 45 cytokines Luminex immunoassays were performed using a 45-plex Luminex assay (Human XL Cytokine Magnetic Luminex Performance Assay, Lot.P276948, R&D Systems, a bio-techne brand) and following the manufacturer’s instructions. The detail containing the 45-plex cytokine assay is presented in **Figure 1A**. Serum concentrations of each cytokine were measured in pg/ml, and positive control samples for each analyte were assayed in parallel to ensure objective results. For the validation stage, single human cytokine Luminex immunoassays of TRAIL (Human TRAIL/TNFSF10 XL Magnetic Luminex Performance Assay, Lot.LUXLM375, R&D Systems, a bio-techne brand) and IL-10 (Human IL-10 Magnetic Luminex Performance Assay, Lot.LUHM217, R&D Systems, a bio-techne brand) were performed on 288 enrolled patients according to the manufacturer’s instructions. All samples were randomized to locate in plates.

### Prostate health index (PHI) score

The prostate health index (PHI) test was performed by the Beckman Coulter DxI800 Unicel Immunoassay system (Beckman Coulter, Shanghai, China) including the measurement of total PSA (tPSA), free PSA (fPSA), and [-2] pro-PSA (p2PSA). The PHI test was used to determine the PHI score according to the formula: $\text{PHI}=\left(\frac{\text{p}2\text{PSA}}{\text{free PSA}}\times \left(\text{tPSA}\right)1/2\right)$ .

### Statistical analysis

The normal distribution of variables was assessed by the Kolmogorov–Smirnov test, while the comparisons of parametric and nonparametric continuous variables were tested by the Student T-test, and the Mann–Whitney U-test. Univariable and multivariable logistic regression analysis was used to determine the association between covariates and AG PCa performed by the “generalized linear model” function with binomial parameters in R. The ROC (Receiver-operating characteristic) curve analysis and AUC (area under the receiver operating characteristic curve) was used to quantify the predictive accuracy of several prediction models for early detection of AG PCa in the PSA grey zone. The best prediction model was graphically presented as a nomogram for clinical use. Decision curve analysis (DCA) was performed by the “ggDCA” (version 1.1) and “rms” (version 6.2) R packages and used to compare the predictive accuracy of prediction models mentioned above. The analysis was performed by R (version 4.0.0) and MedCalc (version 15.2.2).

## Results

Of the 320 registered men in our study, 288 (90.0%) who were within the PSA grey zone and met the study criteria were eventually recruited and formed the training cohort. Among all the samples, 16 cases were excluded due to previous invasive prostate treatment history, 11 were confirmed to exceed the PSA-related grey zone criterion by PHI testing and 5 had no serum cytokine data due to insufficient quantity for measurement. Altogether, 209 (72.6%) men had confirmed a negative biopsy or indolent PCa, and 79 (27.4%) men diagnosed with AG PCa were included. Participants’ detailed clinicopathologic characteristics were shown in **Table 1**. Compared to those low-risk diseases, higher levels of total PSA, p2PSA, PHI, IL-10, TRAIL, and less of fPSA were presented in patients with AG PCa, while the age did not present significance within men with AG PCa and patients with indolent disease.

**Table 1:** Clinical characteristics of the study cohorts within PSA grey zone.

Median (IQR)	All patients	Benign or indolent PCa (GS = 6)	AG PCa (GS ≥ 7)
**Subjects, n**	288	209	79
**age, years**	69 (65 - 73)	70 (65 - 75)	67 (64 - 70)
**tPSA, ng/mL**	7.27 (5.75 - 9.10)	6.99 (5.56 - 8.50)	8.76 (6.85 - 9.85)
**fPSA, ng/mL**	1.10 (0.81 - 1.69)	1.22 (0.90 - 1.91)	0.91 (0.61 - 1.33)
**p2PSA, pg/mL**	15.07 (8.83 - 24.91)	13.97 (8.66 - 23.84)	20.73 (12.33 - 33.67)
**PHI**	36.95 (20.69 - 68.02)	28.75 (17.28 - 48.01)	73.51 (43.73 - 117.19)
**IL - 10, pg/ml**	177.00(147.10 - 203.73)	172.75 (146.76 - 199.02)	191.52 (151.56 - 219.81)
**TRAIL, pg/ml**	46.66 (29.99 - 73.56)	37.29 (25.24 - 55.96)	74.69 (63.05 - 87.65)

Benign, biopsy negative benign prostatic hyperplasia; PCa, prostate cancer; AG, aggressive; GS, Gleason score (biopsy); tPSA, total PSA; fPSA, free PSA; p2PSA, [-2] pro-PSA; PHI, prostate health index, PHI = [(p2PSA/free PSA) × (PSA)½]; IL-10, interleukin 10; TRAIL, TNF-related apoptosis induced ligand; IQR, interquartile range; Data are given as median (IQR), unless otherwise indicated.

Serum cytokines were tested through comprehensive screening by multiplex immunoassay to identify potential candidates relevant to AG PCa (Human XL Cytokine Luminex Performance Panel Premixed Kit, Lot. FCSTM18, R&D Systems, a bio-techne brand). In the discovery stage, a total of 45 candidate serum cytokines were selected to be assessed following the vendor’s recommended protocol in a small cohort consisting of 10 samples from AG PCa or benign prostate diseases patients, which were collected from Xinhua Hospital with institutional approval. We defined the statistical criteria for selecting different levels of serum cytokines using *P* values < 0.05. The heatmap showed the concentration of serum cytokines (**Figure 1A**). In total, we identified 2 candidate biomarkers TRAIL and IL-10 that had significantly different levels between individuals with AG PCa and benign disease and further evaluated them using Luminex single cytokine immunoassays in the 288 sera. The analysis of serum TRAIL and IL-10 levels from AG PCa and BPH or indolent PCa patients, as well as diagnostic scores of tPSA and PHI, were demonstrated in scatterplots (**Figure 1B**), which presented a statistical significance between BPH/indolent PCa and AG PCa patients, though there was no significant correlation between PHI scores and concentrations of cytokines TRAIL and IL-10, respectively (**Figure 1C**).

Univariable and multivariable logistic regressions were then used to screen for independent risk factors and construct prediction models to estimate the probability of AG PCa. The result showed that in univariable logistic regression, PHI-based biomarkers and serum cytokines TRAIL, IL-10 were all associated with AG PCa, while multivariable analysis demonstrated that tPSA, fPSA, PHI, IL-10, and TRAIL were the 5 independent risk factors but age and p2PSA were excluded (**Table 2**).

**Table 2:** Univariable and multivariable logistic regression analysis testing variables as independent risk factors of AG PCa.

	Univariable analysis		Multivariable analysis	
	Odds ratio (95%CI)	*P*	Odds ratio (95%CI)	*P*
**age**	0.95 (0.91 - 0.99)	**0.013**	0.97 (0.92 - 1.02)	0.24
**tPSA**	1.28 (1.14 - 1.43)	**<0.001**	1.47 (1.23 - 1.76)	**<0.001**
**fPSA**	0.45 (0.3 - 0.69)	**<0.001**	0.31 (0.16 - 0.61)	**0.001**
**p2PSA**	1.01 (1 - 1.02)	0.05	0.99 (0.97 - 1.01)	0.184
**PHI**	1.02 (1.01 - 1.02)	**<0.001**	1.02 (1.01 - 1.03)	**<0.001**
**IL - 10**	1.01 (1 - 1.02)	**0.006**	1.01 (1 - 1.02)	**0.024**
**TRAIL**	1.05 (1.04 - 1.06)	**<0.001**	1.05 (1.04 - 1.07)	**<0.001**

Bold values indicate significant difference. AG, aggressive; CI, confidence interval; tPSA, total PSA; fPSA, free PSA; p2PSA, [-2] pro-PSA; PHI, prostate health index, PHI = [(p2PSA/free PSA) × (PSA)½]; IL-10, interleukin 10; TRAIL, TNF-related apoptosis induced ligand.

The ROC curve analysis suggested that the addition of the TRAIL significantly increased the AUC of the model based on PHI alone from 0.797 (95%CI 0.738−0.855, *P*<0.0001) to 0.884 (95%CI 0.845−0.922, *P*<0.0001) in AG PCa prediction. Meanwhile, the highest accuracy (AUC 0.915; 95% CI: 0.880–0.951) was obtained with the introduction of the full risk model that included the tPSA, fPSA, PHI, TRAIL, and IL-10 (**Figure 1D**). Notably, decision-curve analysis (DCA) suggested a superior net benefit of the full risk model over various diagnostic strategies (*P*<0.0001), which indicated a good discriminative capability (**Figure 1E**). DCA also demonstrated that the full risk model could achieve better net benefit than other models between approximately 50% and approximately 75% high-risk threshold. Moreover, PHI + TRAIL model and PHI + IL-10 + TRAIL models were also analyzed on DCA which affirmed the value of including the two serum cytokines in the full risk prediction model. Furthermore, to expand the utility of the full risk model, a nomogram was graphically depicted according to these results (**Figure 1F**). Consistently with the AUC results, the full risk model performed an improvement in a clinical net benefit according to the nomogram for AG PCa within the PSA grey zone.

## Discussion

As one of the promising models for AG PCa early detection, PHI has been regarded as the best non-invasive predictive one for improving diagnostic accuracy and reducing unnecessary biopsies, which outperforms any other PSA-based derivatives. With the introduction of novel serum cytokine-related prediction tools, further improvements emerged in the detective utility of the PHI-based multivariate models for clinically AG PCa within the PSA grey zone (22).

In the present study, we have performed a 45 serum multi-cytokine screening for AG PCa-specific biomarkers and thoroughly analyzed the efficacy of multivariate prediction models incorporating the PHI score. Univariable logistic regression was analyzed for single biomarkers while their various combinations were measured by multivariable logistic regression. Each multivariable model was then fitted to predict AG PCa within the PSA grey zone, then converted to percentage probabilities to show the predictive ability. There was no significant difference in the concentrations of these majority of cytokines between AG PCa patients and indolent/benign cases. However, the authors found markedly elevated concentrations of TRAIL (TNF-related apoptosis induced ligand) and IL-10 (interleukin 10) in PCa (*P*<0.05), while the combination of TRAIL, IL-10, and PHI score showed the best predictive performance for AG PCa. Concomitantly, due to the utmost importance of Gleason score in PCa management, it is the high-grade disease (Gleason≥7) that dictates the need for aggressive treatment options, thus we have included this important metric throughout the study and concluded that the levels of serum TRAIL and IL-10, are clinically useful for the prediction of Gleason≥7 AG PCa in PHI-based multivariable prediction models.

Consistent with the results of recent studies the risk factor immune response was found to be related to aggressive prostate cancer progression and resistance to drug treatment (24–26), which may further direct urologists for grey zone aggressive prostate cancer early risk stratification. Functioning as one of the tumor necrosis factor ligand family, TRAIL has been reported to induce apoptosis in tumor cells preferentially. Recent studies have reported that targeting TRAIL is a promising anticancer therapy for prostate cancer, and harnessing TRAIL-induced apoptosis pathway for immunotherapy has attracted attention rapidly as a cancer treatment target (27, 28). However, the existing reports on TRAIL are focusing on the function of tumor cell apoptosis induction, and no study on diagnostic biomarker value for PCa has been proposed. Thus, a gap lies in understanding the key switches that control TRAIL signaling in tumorigenesis. Moreover, IL-10 is a major immune-regulatory cytokine that has pleiotropic effects in immunoregulation and inflammation including profound anti-inflammatory and limiting excessive tissue disruption capability (29). A recent study suggested that the AR signaling in THP-1 and monocyte-derived macrophages followed by IL-10 upregulation may support PCa invasiveness, which might be a possible explanation for high levels of serum IL-10 in AG PCa (30). Another study by Faupel-Badger et al. also showed a correlation between IL-10 and PCa risk and grade of disease (31). Compared with the tissue sample from active surveillance patients, the acquisition of blood samples is easier while extra interference to PHI related biomarkers can avoid. With regards to the critical role of the PHI model and potential predictive utility of serum TRAIL and IL-10 in the PCa tumorigenesis and the progression, the combination of biomarkers above for the AG PCa risk prediction may become a promising direction for PCa early detection in the future. As mentioned above, understanding how to early predict the grey zone AG PCa occurrence to enable more treatment opportunities and improve survival benefit is of great importance in daily clinical work.

Since this is the first study aiming to construct PHI-based multivariable prediction model by serum multi-cytokines screening to improve prostate cancer risk assessment, we acknowledged that several limitations and biases might exist in the present research. Among them, limitations by a relatively small single-center cohort and ethnically homogenous study population have potentially affected the applicability of the new predictors for external validation. Besides, due to the patient willingness-based recruitment, selection bias was unable to be excluded. Hence, more work and further validation in multicenter, large-sample cohorts are urgently needed.

## Conclusions

Our data suggest a significant advantage for the PHI-based multivariate combinations of serum TRAIL, IL-10 comparing with PHI or other serum-derived biomarkers alone in the detection and risk stratification of grey zone AG PCa. Meanwhile, this assay, which does not require invasive or special handling, can be easily applied as a basic clinical workflow just like the routine PSA screening. Furthermore, in line with the recent studies, we also recommend using multivariate combination predictive models involving prostate volume parameters, PHI, and serum cytokines due to a possible improved diagnostic efficacy for AG PCa early detection within the PSA grey zone.

## Data availability statement

The raw data supporting the conclusions of this article will be made available by the authors, without undue reservation.

## Ethics statement

The studies involving human participants were reviewed and approved by The Ethics Committee of Xinhua Hospital affiliated to Shanghai Jiao Tong University School of Medicine approved this study (XHEC-C-2019-113-2). The patients/participants provided their written informed consent to participate in this study.

## Author contributions

YY, XC and JD contributed to the conception and design of the manuscript. JL and YQ contributed to the acquisition of data. HC and YS contributed to the manuscript writing. JZ and YW contributed to the data management and analysis. BS and FQ contributed to the manuscript editing. All authors contributed to the article and approved the submitted version.

## Funding

The presented work was supported by Xinhua Hospital Clinical Innovation Fund (grant no.19XHCR01A) and Clinical Research Plan of SHDC (grant no. SHDC2020CR4034).

## Acknowledgments

We would like to thank all the participants and coordinators who joined this study.

## Conflict of interest

The authors declare that the research was conducted in the absence of any commercial or financial relationships that could be construed as a potential conflict of interest.

## Publisher’s note

All claims expressed in this article are solely those of the authors and do not necessarily represent those of their affiliated organizations, or those of the publisher, the editors and the reviewers. Any product that may be evaluated in this article, or claim that may be made by its manufacturer, is not guaranteed or endorsed by the publisher.

## Abbreviations

TRAIL, TNF-related apoptosis induced ligand; IL-10, interleukin 10; PHI, prostate health index model; PCa, prostate cancer; PSA, prostate specific antigen; DRE, digital rectal examination; AG, aggressive; BPH, benign prostatic hyperplasia; tPSA, total prostate specific antigen; fPSA, free PSA; p2PSA. [-2] pro-PSA; AUC, area under curve; ROC, receiver operating characteristic curve; DCA, decision curve analysis; GS, Gleason Score.
